# Age-related network connectivity pattern changes are associated with risk for psychosis

**DOI:** 10.1192/j.eurpsy.2022.805

**Published:** 2022-09-01

**Authors:** R. Passiatore, L. Antonucci, T. Deramus, L. Fazio, G. Stolfa, I. Andriola, M. Sangiuliano, M. Altamura, A. Saponaro, F. Brudaglio, A. Carofiglio, T. Popolizio, F. Sambataro, G. Blasi, A. Bertolino, V. Calhoun, G. Pergola

**Affiliations:** 1 University of Bari Aldo Moro, Basic Medical Science, Neuroscience And Sense Organs, Bari, Italy; 2 Georgia State University, Tri-institutional Center For Translational Research In Neuroimaging And Data Science (trends), Atlanta, United States of America; 3 University of Bari Aldo Moro, Department Of Education, Psychology And Communication, Bari, Italy; 4 University Hospital, Psychiatric Unit, Bari, Italy; 5 University of Foggia, Department Of Clinical And Experimental Medicine, Foggia, Italy; 6 ASL Brindisi, Department Of Mental Health, Brindisi, Italy; 7 Department of Mental Health, Asl Barletta-andria-trani, Andria, Italy; 8 Department of Mental Health, Asl Bari, Bari, Italy; 9 IRCCS Casa Sollievo della Sofferenza Hospital, Neuroradiology Unit, S. Giovanni Rotondo, Italy; 10 University of Padova, Department Of Neuroscience, Padova, Italy; 11 Lieber Institute for Brain Development, Johns Hopkins Medical Campus, Baltimore, United States of America

**Keywords:** fMRI, Risk for psychosis, Independent Component Analysis, Neurodevelopment

## Abstract

**Introduction:**

Psychosis onset typically occurs during adolescence or early adulthood, coinciding with the latest stage of brain maturation. Alterations in brain functional connectivity (FC) accompany the emergence of psychiatric symptoms and cognitive impairments. Thus, age-related FC changes may be informative regarding psychosis onset.

**Objectives:**

We defined neurotypical age-related FC trajectories and hypothesized that FC of individuals at familial and clinical high risk (HR) for psychosis deviates from FC of neurotypical controls (NC).

**Methods:**

We analyzed two independent cohorts, of (a) 356 early adult NC (yNC; age=22±2y, m:f=149:207), and 127 mature adult NC (aNC; age=38±7y, m:f=79:48), and (b) 92 yNC (age=22±2y, m:f=34:58), 33 aNC (age=36±6y, m:f=21:12), 38 early HR adults (age=20±3y, m:f=18:20). We acquired fMRI data from multiple scans (resting-state, working memory, episodic memory, and implicit emotion processing). FC was obtained by computing Pearson’s correlations between time-courses of every independent component (IC) defined by an Independent Component Analysis approach (NeuroMark). Age-varying components of interest (yNC/aNC differences on FC based on linear mixed effect regressions) were tested for differences between HR and yNC through the Wilcoxon rank-sum test.

**Results:**

showed age-related FC differences (yNC/aNC) in a set of 17 IC pairs (p_FDR_<0.05). HR showed increased FC within a network including dorsolateral and medial prefrontal cortices, and sensorimotor cortex, while decreased FC between cerebellum and the parietal and visual cortices, compared with yNC (p_FDR_<0.05). HR showed no significant difference compared with aNC (p_FDR_>0.05).

**Conclusions:**

This study tested FC alterations associated with the risk for psychosis and highlighted the relationship between psychosis and potentially altered brain functional processes.

**Disclosure:**

No significant relationships.

